# Using item response theory to identify key symptoms of insomnia in a sample of university students with probable eating disorders

**DOI:** 10.1007/s40519-024-01679-z

**Published:** 2024-07-28

**Authors:** Kara A. Christensen Pacella, Brianne N. Richson, Nicole A. Short, Angeline R. Bottera, Leah A. Irish, Victoria L. Perko, Kelsie T. Forbush

**Affiliations:** 1 Department of Psychology, University of Nevada, Las Vegas, Las Vegas, NV USA; 2 Center for Biobehavioral Research, Sanford Research, Fargo, ND USA; 3 Department of Psychology, University of Kansas, 1415 Jayhawk Blvd, Lawrence, KS 66045 USA; 4 Department of Psychology, North Dakota State University, Fargo, ND USA; 5 ReefPoint Group, LLC, Tysons Corner, Virginia USA

**Keywords:** Insomnia, Item response theory, University students, Eating disorders

## Abstract

**Purpose:**

Researchers have theorized that interactions between appetitive and circadian disruptions result in increased eating disorder (ED) symptoms and insomnia. However, it is unclear how specific insomnia symptoms present among people with EDs and if the latent structure of insomnia in this population is similar to that of people with insomnia disorder.

**Methods:**

We conducted a secondary analysis of data collected on ED and insomnia symptoms using a subset of students (*N* = 547; 79.52% female) with probable EDs at a large Midwestern American university. Item response theory (IRT) for polytomous items was performed to identify item difficulty, discrimination, and information parameters for the Insomnia Severity Index (ISI). IRT parameters were compared to those established in a 2011 study of people diagnosed with insomnia disorder by Morin and colleagues.

**Results:**

Clinically significant insomnia symptoms were common among students with ED pathology and symptom endorsement for each ISI item ranged from 40.77 to 86.65%. ISI items assessing insomnia-related impairment and distress showed better discriminative capacities and had higher item information than items assessing sleep behavior alterations (i.e., difficulties falling asleep, difficulties maintaining sleep, waking too early). Item discrimination was largely similar among the ED sample compared to previous IRT analyses in an insomnia disorder sample.

**Conclusion:**

Insomnia symptoms are common among university students with probable EDs and similar to those reported by people with insomnia disorder. When considering insomnia assessment, items assessing sleep behaviors alone are likely inadequate to provide information about insomnia severity among people with EDs.

**Level V:**

Evidence obtained from a cross-sectional descriptive study.

Emerging research suggests a bidirectional association between sleep and eating behaviors which, over time, may lead to the development and maintenance of clinically significant psychopathology, such as insomnia and eating disorders (EDs), e.g., [1, 2]. Further evaluation of insomnia in EDs is warranted in light of research suggesting that insomnia influences the development of internalizing psychopathology [3], places individuals at greater risk for relapse following psychological treatment, e.g., [4], and is associated with poorer quality-of-life and health [5].

Several studies have found a positive association between insomnia severity and disordered-eating symptoms in college student and/or young adult populations [6, 7], and female college students with EDs are significantly more likely to report clinically significant insomnia symptoms than those without EDs (25–30% vs. 5%, respectively) [8]. In addition to causing distress and impairment, insomnia symptoms are linked to poorer ED treatment outcomes, e.g., [9]. Recent literature points to a likely bidirectional relationship between insomnia and ED symptoms, broadly; that is, insomnia symptoms may worsen ED behaviors, such as binge eating and restricting, which further exacerbate insomnia symptoms [1, 2]. This positive feedback loop may result in continued cognitive and behavioral disruptions, maintaining both insomnia and ED symptoms. For example, sleep restriction and altered sleep timing may result in increased craving and consumption of palatable food [10–13], potentially elevating risk for binge eating. Further, binge eating is more likely to occur in the late afternoon or evening [2, 14] and may consequently delay sleep onset [1]. Therefore, insomnia symptoms may increase risk for binge eating via disruptions to daily appetite rhythms, and binge eating may increase risk for insomnia symptoms by varying sleep–wake timing [15].

Comparatively less is known about how different insomnia complaints (e.g., difficulty falling asleep, impairment, distress) present among people with EDs. This question is important because certain insomnia presentations (e.g., sleep onset insomnia, sleep maintenance insomnia, early morning awakening insomnia) have been differentially associated with insomnia severity and daytime functioning [16] and patterns of comorbidity, including anxiety, depression, substance use, and hypnotic medication use, e.g., [17]. In a study evaluating prevalence of insomnia, Kim et al. [18] found that in a sample of four hundred women with EDs, difficulty initiating sleep was the most common sleep behavior complaint (32.6%), followed by difficulties maintaining sleep (17.2%), and waking up too early (8%). Consistent with the cognitive model of insomnia [19], individuals with EDs report elevated levels of worry and rumination [20–22] and demonstrate patterns of increasing negative affect across the day [23–25], which may ultimately maintain insomnia symptoms by increasing nighttime arousal. However, only two prospective longitudinal studies examined how ED problems were associated with difficulties initiating and maintaining sleep. First, a study by Nagata et al. [26] found that among people with a probable ED diagnosis, ED behaviors were linked to difficulty initiating asleep only among people with restrictive eating. Another study by Bos et al. [27] found that global disordered eating pathology was associated with difficulties initiating and maintaining sleep in Colombian university students a year later. However, the literature examining insomnia symptoms has tended to focus on sleep behavior alterations (e.g., difficulty falling or staying asleep) with limited assessment of sleep-related impairment and distress, which is important for understanding the consequences of sleep behavior alterations and a critical component to identifying clinically significant insomnia problems. Thus, there remains a gap in our knowledge of how impairment and distress related to insomnia presents among people with EDs.

Taken together, the literature suggests that specific insomnia complaints may vary among people with EDs although there is limited knowledge about impairment and distress. Furthermore, it is unclear whether the endorsement of specific insomnia symptoms (e.g., sleep behavior alterations, impairment) is associated with increased insomnia severity in the context of EDs or if sleep behavior alterations and daytime dysfunction are interchangeable in their associations with the latent construct of insomnia. If they are not interchangeable, then it would suggest that research approaches that do not consider behaviors, distress, and impairment are incomplete for understanding insomnia among people with EDs. Furthermore, identification of items that best capture the latent construct could inform best practices for brief screenings for sleep problems among people with EDs. Thus, the goal of this study is to better characterize the frequency of specific insomnia symptoms (encompassing impairment and distress) among people with EDs and provide further analysis of how these symptoms relate to the overall latent construct of insomnia in this population.

## Item response theory and symptom-level insomnia problems

Item response theory (IRT) can be used to understand insomnia symptoms’ association with latent insomnia severity. In this study, we performed IRT with the commonly used Insomnia Severity Index (ISI [28]). Each ISI item is weighted equally to contribute to the total score, which assumes that endorsement of individual items is interchangeable in contributing to overall current insomnia severity; however, as reflected by IRT analyses, responses to certain items may be more indicative of clinically significant insomnia than others. Further, based on an item’s discriminative ability, certain items may yield more versus less specific information about underlying severity than others. For example, an item with low discrimination may be endorsed widely irrespective of severity, meaning that it may not be particularly useful for understanding severity.

Although the ISI is widely used for insomnia screening and has demonstrated strong psychometric properties including internal consistency, criterion validity, concurrent validity and predictive validity [29], to date there has only been one study examining its properties using IRT. Morin et al. [35] examined the performance of the ISI in a sample of people with insomnia. They found that difficulty falling asleep and waking up too early showed poor discriminative capacity, whereas all other items exhibited adequate to excellent discriminative capacity. In other words, difficulty falling asleep and waking up too early were not the best indicators of overall insomnia severity. A different possibility, however, was that these two items were not reflective of insomnia severity in the context of the restricted range of insomnia symptoms represented in a sample of individuals known to have insomnia disorder, but may provide more information in an unselected sample.

Although the study by Morin et al. [35] provided initial information about the ISI item performance, a limitation is that the authors did not evaluate item difficulty (i.e., degree of severity associated with a given item response) or item information (degree of measurement error). Furthermore, it is unclear if performance on the ISI items in a sample of people with diagnosed insomnia disorder, as in Morin et al. [35] is similar to that of a sample of people with EDs, whose sleep problems may differ from typical presentations of insomnia disorder.

Thus, for the present study, we analyzed responses on the ISI among university students with probable EDs. First, we examined the prevalence of probable insomnia in the sample and the rate of endorsement of the seven symptoms assessed by the ISI to help characterize the nature of insomnia problems in this population. Next, we conducted an IRT analysis of the ISI to determine which ISI items were most reflective of severe, likely clinically significant, insomnia and how varying levels of insomnia severity were associated with responses for each ISI item. We hypothesized that the ISI items in the ED sample would perform similarly to those in the original IRT analysis of people with insomnia disorder by Morin et al. [35].

## Methods

### Participants

The sample consisted of 547 university students with a probable ED (*M*_age_ = 23.10 years, SD = 6.76). The majority of the sample met criteria for other specified feeding and eating disorder (*n* = 383, 70.02%), followed by bulimia nervosa (*n* = 130, 23.77%), binge eating disorder (*n* = 24, 4.39%) and anorexia nervosa (*n* = 10, 1.83%). The racial composition of the sample was 82.27% White, 6.40% multiracial, 5.48% Asian or Pacific Islander, 3.66% Black, and 1.28% American Indian or Alaska Native (0.91% of participants did not identify their race), with 10.24% of the sample also reporting their ethnicity as Hispanic. The majority of participants reported they were women (79.52%); however, 15.72% reported they were men and 4.75% reported belonging to a minoritized gender group (e.g., transgender man, transgender woman, non-binary). Of note, cisgender was not specified in the question responses, therefore the “woman” or “man” categories were not exclusive to cisgender individuals. The mean BMI for the sample was 26.80 kg/m^2^ (SD = 7.15) (Table 1).

**Table 1:** Characteristics of the sample

	*M* (SD)	Range
Age	23.10 (*6.76*)	18–72
Body mass index	26.80 *(7.15)*	14.12–66.17
Insomnia severity	12.04 *(5.79)*	0–28
Eating disorder-related impairment	26.58 *(8.20)*	16–48

	%	*n*
Gender		
Man	15.72	86
Minoritized gender identity	4.75	26
Woman	79.52	435
Ethnicity		
Hispanic	10.24	56
Non-Hispanic	89.76	491
Years of full-time higher education		
Less than 1 year	4.20	23
One year	16.45	90
Two years	25.59	140
Three years	24.50	134
Four years	10.24	56
Five years	8.96	49
Six years or more	10.05	55
Race		
American Indian or Alaskan Native	1.28	7
Asian or Pacific Islander	5.48	30
Black or African American	3.66	20
Multiracial	6.40	35
White	82.27	450
Did not indicate	0.91	5
ED diagnosis		
Anorexia nervosa	1.83	10
Binge eating disorder	4.39	24
Bulimia nervosa	23.77	130
Other specified feeding or eating disorder	70.02	383

Table contains the demographic characteristics of the analytic sample (*N* = 547). Due to missing values, sample size for age (*n* = 527) differed. Minoritized gender identity included participants who identified as transgender men or transgender women, gender non-conforming, genderqueer, or another non-cisgender identity; however, the question response options to gender were such that cisgender was not specified, therefore the “woman” or “man” categories are not exclusive to cisgender individuals

### Procedures

Our local Institutional Review Board approved all study procedures and participants provided informed consent. The stated purpose of the survey was to evaluate disordered-eating behaviors and body image concerns in the university student body; therefore, all students were encouraged to participate, regardless of whether they perceived themselves as having issues related to body image or eating. Participants were invited to take the REDCap survey through a mass email sent to all students (*N* = 17,751 students), except freshmen, in September 2020. Freshmen were not included, as our team surveyed them in a later wave in the academic year. The overall survey response rate was 10.084% (*N* = 1790), which included 1243 students without a probable ED (69.44%) and 547 students with a probable ED (30.56%). For this study, we included only students who screened positive for an ED. Race and ethnicity within the study sample was largely similar to the overall demographics of the university [30].

### Measures

#### Questionnaires

Demographics: Participants answered questions regarding age, race, ethnicity, gender, student status (i.e., undergraduate, graduate, non-degree seeking), and number of completed years of post-high school education.

The Clinical Impairment Assessment (CIA) [31]: The CIA is a 16-item self-report measure that assesses the impact of ED psychopathology on functioning over the past 28 days. The CIA uses a four-point scale, ranging from zero to three. A global score (0–48) is calculated by summing all items, with higher scores indicating greater impairment. Psychometric research on the CIA suggests the use of a cut-off score of 16 for distinguishing ED cases [32]. In this sample, internal consistency of CIA items was good, *α* = 0.88. The CIA was used to generate probable ED diagnoses.

Eating Disorder Diagnostic Scale 5 (EDDS) [33]: The Eating Disorder Diagnostic Scale is a 22-item self-report diagnostic measure that assesses *DSM-5* symptoms of EDs. An additional question assessing subjective binge-eating episodes was added to the questionnaire to permit the assessment of purging disorder and compensatory behaviors related to subjective binge eating (“How many times per month on average over the past 3 months did you feel that your eating was out of control (that you could not stop once you started) after eating a SMALL or NORMAL amount of food (e.g., after eating a few standard-size cookies or a standard-size meal)?”). Furthermore, on the symptom frequency questions, the original EDDS had a ceiling of 12+ episodes per month. We extended this to 16+ to allow for increased variability in severity. The EDDS was used to generate probable ED diagnoses. Online surveys evaluating ED prevalence have been widely used among university students, notably among the nationwide multi-institution annual Healthy Minds Study [34].

Insomnia Severity Index (ISI) [28]: the ISI is a seven-item measure that assesses insomnia severity and impairment over the past two weeks. The first three questions evaluate different sleep behavior alterations (i.e., difficulty falling asleep, difficulty remaining asleep, and waking up too early). The other four questions evaluate satisfaction with sleep, how noticeable the sleep problem is to others in impairing quality-of-life, worry/distress related to the sleep problem, and interference with daily functioning. The ISI uses a five-point scale from zero to four; all items are summed to create a total score, such that higher scores indicate greater insomnia severity. A score of ten on the ISI has been identified as an appropriate cutoff for detecting probable insomnia case status in a community sample, although it should be noted that this is not synonymous with insomnia disorder diagnosis [35], as the ISI does not provide information about the duration of symptoms or potential rule-out diagnoses or conditions. The ISI has demonstrated adequate reliability and validity [28]. In this sample, internal consistency of ISI items was good, *α* = 0.86.

#### Probable ED diagnoses

Probable ED diagnosis was established using the diagnostic coding from the EDDS combined with scores from the CIA (see [57]). Behavioral frequencies were used to diagnose anorexia nervosa, bulimia nervosa, and binge eating disorder. To capture other specified feeding and eating disorder diagnoses, individuals with sub-threshold ED symptoms (e.g., significant ED behaviors, but number of episodes below frequency) who also reported ED-impairment at or above the CIA cut-off value of 16 were classified as having a probable ED.

### Statistical analysis

Survey responses were visually inspected for invalid responding (e.g., impossibly low BMI, inappropriate write-in answers) by trained research assistants prior to initiating analysis. The first author reviewed and removed potentially invalid responses (*n* = 1) and duplicate records (*n* = 38). Maximum likelihood multiple imputation procedures, with the R package “Amelia II” [36] were used to address missing ISI data (8.14% across seven ISI items). We conducted five imputations and averaged to create a final imputed value for each missing response.

To examine rates of endorsement for each ISI item, we considered a response of two or greater (i.e., “moderate” or “somewhat”) on a 0–4 scale as present. We reported the presence of probable insomnia case using an ISI cut-off score of 10 [35].

To examine the degree of latent insomnia severity indicated by each ISI item, we used unidimensional, polytomous IRT models known as ‘graded response’ models [37] using the R package ‘ltm’ [38]. Unidimensionality refers to a shared construct underlying a set of items, or at least that one predominant construct emerges if items are not best represented by a single construct [39]. Here, the single construct being measured was insomnia severity. Unidimensionality was tested by conducting a confirmatory factor analysis (CFA) model with a robust diagonally weighted least squares (WLSMV) estimator for ordinal data, in which all seven ISI items were required to load onto one factor, using the R package ‘lavaan’ [40]. ISI items were kept in their polytomous Likert-scale format.

First, we conducted a graded response model that produced unique ‘difficulty’ parameters for each ISI item. Here, item difficulty can be conceptualized as severity reflected by a given response on an item. A higher difficulty parameter for a given response category requires a higher latent trait level for endorsement; difficulty parameters for each of an item’s response categories indicated the level of latent insomnia at which there was a 50%-percent chance of someone with that degree of insomnia endorsing a more severe response category [41]. This first model held item discrimination (the extent to which the probability of response-category endorsement changes across levels of the latent construct) constant across items. Second, we conducted a graded response model that estimated both unique ‘difficulty’ and ‘discrimination’ parameters for each ISI item. This approach thus allowed for: (1) determination of how ISI responses related to degree of insomnia severity (item difficulty) and (2) determination of how response likelihood changed at different degrees of insomnia severity (item discrimination, with high discrimination reflecting better differentiation between different levels of insomnia). Nested model comparison determined which of the two models was superior to yield confidence in which model best characterizes how ISI item responses vary across latent insomnia levels. Finally, item information curves (with information corresponding to discrimination and with higher information reflecting lower measurement error), were generated; these curves indicate how each item’s provided information changes across the range of the latent trait. The statistical plan for this study was not pre-registered.

## Results

### Insomnia symptom characteristics

Overall, insomnia problems were common in this sample, with 65.8% of students reporting total ISI scores at or above the cutoff of 10 for probable insomnia case (*M* = 12.04, SD = 5.79). Dissatisfaction with sleep (86.65%) was the most endorsed symptom, followed by difficulty initiating sleep (65.45%), daily interference resulting from sleep problem (63.62%), difficulty maintaining sleep (50.09%), worry/distress about sleep (47.35%), how noticeable sleep problem is to others (43.88%), and waking up too early (40.77%) (Table 2).

**Table 2:** Item-level endorsement rates on ISI

	0	1	2	3	4	Symptom endorsed
1. Difficulty initiating sleep	12.98%	21.57%	35.28%	21.02%	9.14%	65.45%
2. Difficulty maintaining sleep	23.77%	26.14%	29.98%	12.98%	7.13%	50.09%
3. Waking up too early	34.00%	25.23%	23.22%	10.79%	6.76%	40.77%
4. Dissatisfaction with sleep	1.10%	12.25%	36.93%	38.76%	10.97%	86.65%
5. How noticeable sleep problem is to others	27.06%	29.07%	26.87%	10.60%	6.40%	43.88%
6. Worry/distress about sleep	19.56%	33.09%	30.53%	12.07%	4.75%	47.35%
7. Daily interference from sleep	11.33%	25.05%	33.46%	20.66%	9.51%	63.62%

A symptom was considered endorsed if participant rated it as a two or above

#### IRT models

To test the IRT assumption of unidimensionality, a one-factor CFA model with a WLSMV estimator indicated that ISI items were represented by one predominant factor based on factor loadings > 0.65 [42], except for one item (item 3) that demonstrated a relatively more moderate loading of 0.458. All factor loadings were significant at *p* < 0.001. Fit indices of CFI = 0.958, TLI = 0.937, and SRMR = 0.067 were overall favorable despite a significant Chi-square statistic (*χ*^2^ = 239.246 [14], *p* < 0.001). Though RMSEA was poor (RMSEA = 0.172), RMSEA favors large models both in terms of degrees of freedom and number of variables [43, 44], which indicates that poor RMSEA may be due, in part, to the relative simplicity of the model (i.e., only one factor represented by 7 items). On average across ISI item indicators, the latent insomnia factor explained 56.4% of the variance. Overall, results support the presence of a predominant latent insomnia factor represented by the ISI items, suggesting suitable unidimensionality for IRT models.

Likelihood ratio testing suggested that the graded response model with unique difficulty and discrimination parameters for each item represented a significant improvement over the model in which only difficulty was uniquely estimated (*p* < 0.001). These results suggest that ISI items are best characterized by a model in which both item difficulty and item discrimination vary across items. Thus, we report and interpret only the non-constrained graded response model.

#### Difficulty parameters

Difficulty parameters across item response categories for each item are displayed in Table 3. Items 1 and 2 (difficulty initiating sleep and difficulty maintaining sleep, respectively) performed very similarly across the latent insomnia spectrum. As latent insomnia increased, there was an increase in probability of these two items being responded to with the next increasingly severe response category rather than a less severe response category. This interpretation of the performance of items 1 and 2 in the sample is evidenced by the somewhat overlapping response curves for each response category. Item 2 was slightly more ‘difficult’ (i.e., more reflective of greater insomnia severity for each response category) than item 1.

**Table 3:** IRT ISI graded response model item parameters

	Difficulty: 0	Difficulty: 1	Difficulty: 2	Difficulty: 3	Discrimination
1. Difficulty initiating sleep	− 1.563	− 0.590	0.651	1.793	1.774
2. Difficulty maintaining sleep	− 1.123	− 0.042	1.225	2.359	1.420
3. Waking up too early	− 1.053	0.568	2.609	4.317	0.679
4. Dissatisfaction with sleep	− 2.559	− 1.344	0.053	1.650	2.272
5. How noticeable sleep problem is to others	− 0.673	0.200	1.195	1.949	2.629
6. Worry/distress about sleep	− 0.900	0.069	1.053	1.809	3.804
7. Daily interference from sleep	− 1.374	− 0.365	0.616	1.485	2.979

Each response category’s difficulty threshold is the level of latent insomnia at which there was a 50% chance of someone with that given insomnia severity responding to the item with a higher severity response category. Thus, no difficulty threshold is produced for the most severe response category (a response of ‘4’ on the ISI)

**Fig. 1:**
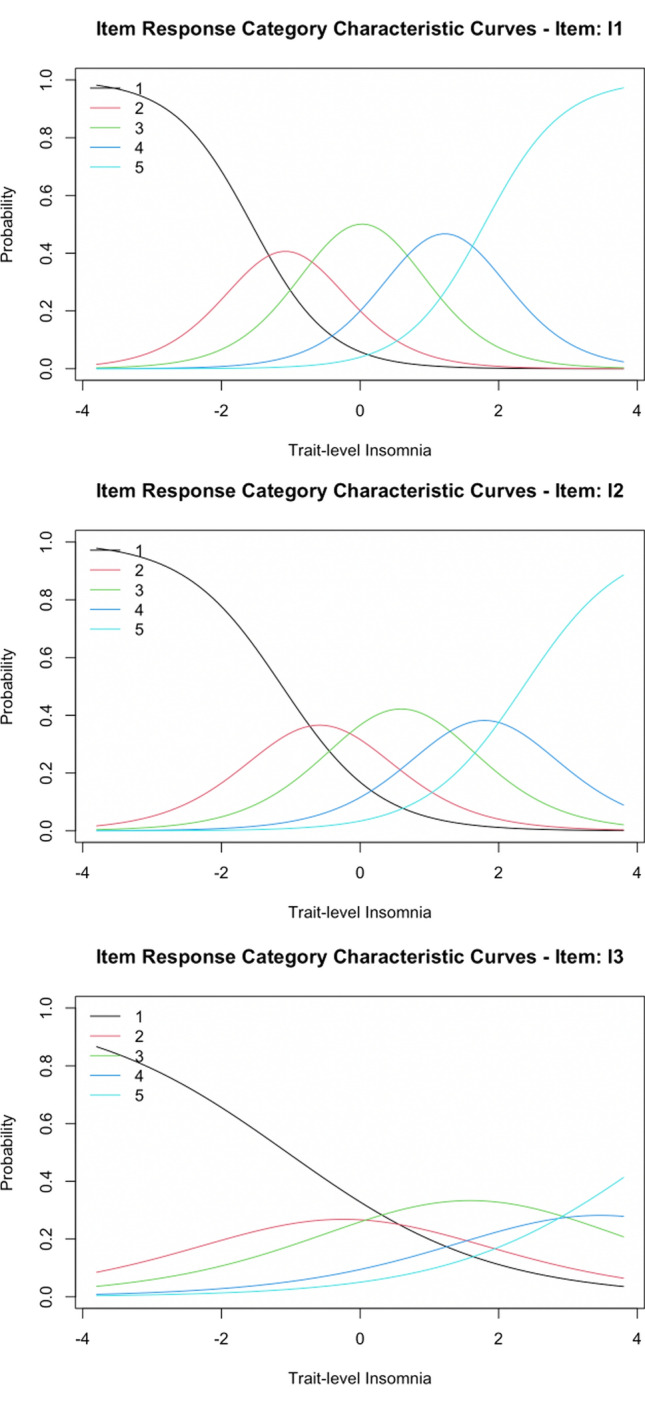
ISI sleep-parameter alteration item response category characteristic curves. Response category characteristic curves for sleep behavior alteration items are displayed above. I1: difficulty initiating sleep; I2: difficulty maintaining sleep; I3: waking up too early

**Fig. 2:**
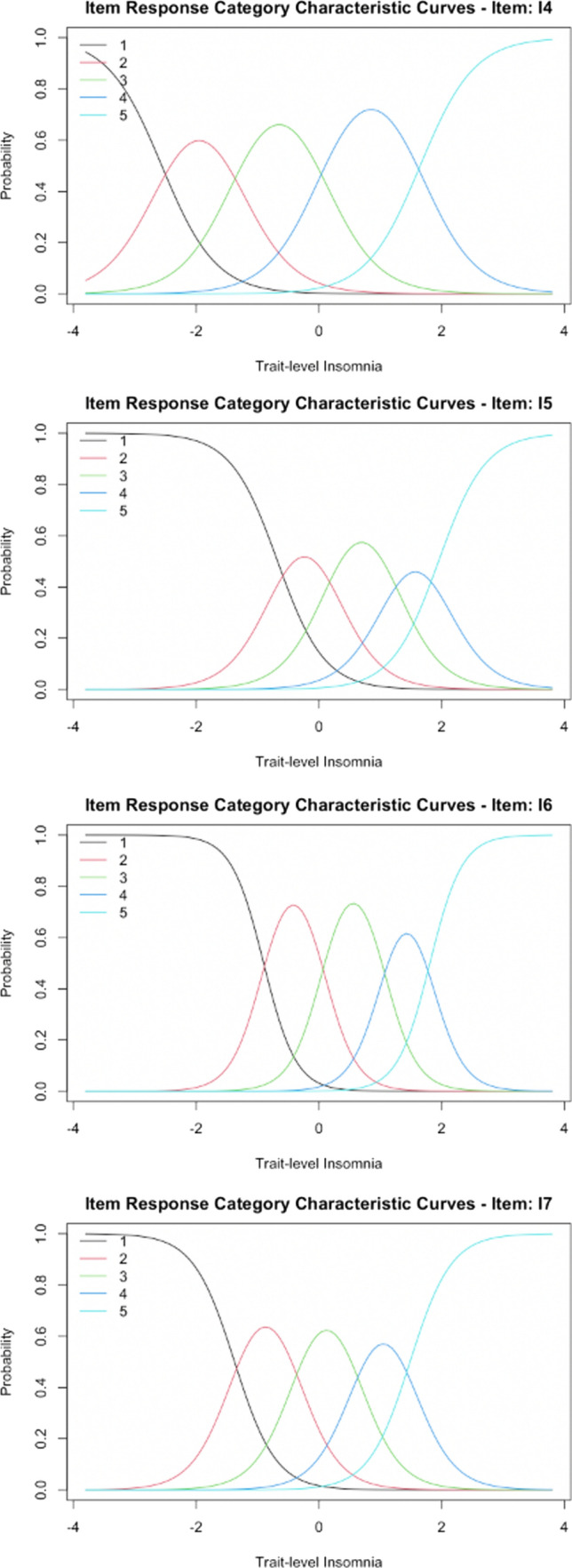
ISI impairment item response category characteristic curves. Response category characteristic curves for insomnia impairment-related items are displayed above. I4: dissatisfaction with sleep; I5: noticeability of sleep problems to others; I6: worry/distress about sleep; I7: daily interference from sleep

Item 3 (waking up too early) was more ‘difficult’ than both items 1 and 2. Across all ISI items, endorsement of the most severe response options for item 3 was associated with comparably greater latent insomnia than the most severe response options for other items. Likewise, as latent insomnia increased, a response indicating no problems waking up too early (item 3) became markedly less likely (Fig. 1). However, milder or moderate problems waking up too early were endorsed relatively indiscriminately across a wide range of the latent insomnia spectrum (as evidenced by very wide, often overlapping item 3 response curves). Thus, degree of overall insomnia needed to be very high for a response in the most severe response categories for waking up too early to be even somewhat likely. In summary, item 3 was a general marker of insomnia in the sample, as endorsement of this symptom occurred across the latent insomnia severity spectrum. Because the endorsement of mild or moderate difficulties with waking up too early was common, item 3 was not a good indicator of overall insomnia severity except for those with very severe insomnia (Fig. 2).

Overall, relative to other items, the item assessing dissatisfaction with sleep (item 4) was reflective of lower latent insomnia severity across item response options. Consistent with the conceptual intent of this item, low latent insomnia levels tended to yield a greater probability of endorsing being very satisfied or satisfied with current sleep. Relative to all other items, item 4 also required comparatively lower levels of insomnia to endorse being dissatisfied or very dissatisfied with current sleep. Considering other dysfunction and impairment items, item 5 (noticeability of sleep problem to others) was the item associated with the highest latent insomnia severity, followed by item 6 (worry/distress about sleep). Item 7 (daily interference from sleep) was also somewhat lower difficulty across response options.

#### Discrimination parameters

Overall, the most discriminating item was item 6 (worry/distress about sleep). In other words, item 6 could best distinguish among people with different levels of latent insomnia severity based on their responses. Item 5 (How noticeable sleep problem is to others) and item 7 (daily interference from sleep) were also more discriminating than other items (Table 3). Item 4 (dissatisfaction with sleep) was less discriminating than other dysfunction and impairment-related items. Other authors have noted that discrimination parameters often fall between 0.5 and 2 [45]; all four of these items demonstrated discrimination parameters > 2, suggesting that all four items had high discriminative ability across the items’ different response categories [46]. In other words, item response categories for these four items functioned well and as intended, such that increasingly severe insomnia was robustly associated with increasing probability of endorsing a more severe response category.

In contrast, items measuring sleep behaviors (items 1–3 measuring difficulty initiating sleep, difficulty maintaining sleep, and waking up too early) yielded relatively lower discrimination parameters compared to all remaining items. Item 3 (waking too early) was particularly poor at discriminating across latent insomnia severity levels relative to all other items (discrimination parameter = 0.679; close to the common lower bound of discrimination parameters of 0.5 cited previously). This result is consistent with the item difficulty results described previously. Specifically, below-average latent levels of insomnia yielded a high probability of reporting no problems with waking too early on item 3, whereas average or greater levels of latent insomnia largely did not greatly change the probability of responding with a given affirmative endorsement category for this symptom (Table 3). Items 1 (difficulty initiating sleep) and 2 (difficulty maintaining sleep) demonstrated similar discrimination parameters and were more discriminating than item 3 (waking too early). Specifically, far below average or far above average levels of latent insomnia yielded highly probable responses of ‘none’ or ‘very severe’, respectively, for difficulty initiating sleep and difficulty maintaining sleep. In contrast, items 1 (difficulty initiating sleep) and 2 (difficulty maintaining sleep) were less discriminating for individuals not at the extremes of the latent insomnia distribution in the sample.

#### Item information

**Fig. 3:**
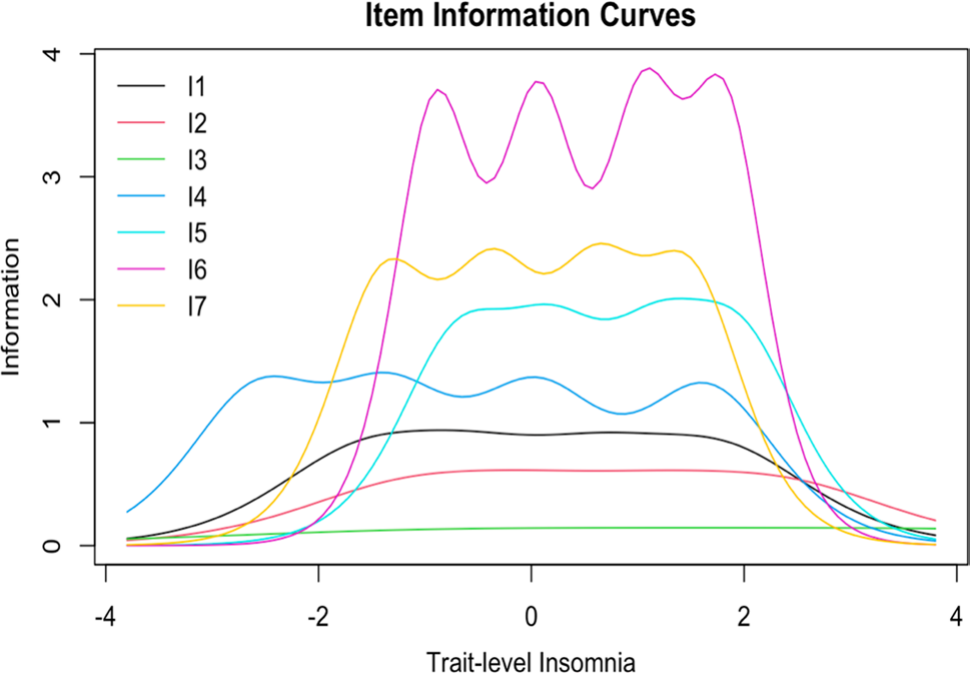
ISI item information curves. Item information curves for each of the seven ISI items are displayed above. I1: difficulty initiating sleep; I2: difficulty maintaining sleep; I3: waking up too early; I4: dissatisfaction with sleep; I5: noticeability of sleep problems to others; I6: worry/distress about sleep; I7: daily interference from sleep

Per item information curves (Fig. 3), item 6 (worry/distress about sleep) provided the highest amount of information, notably for a large range of latent insomnia severity. Item 7 (daily interference from sleep) provided the second highest amount of information for a similarly large range of latent insomnia severity, although was notably less informative at higher levels of severity compared to both items 5 and 6 (worry/distress about sleep) and slightly less informative at higher levels of severity than remaining items.

Consistent with item discrimination results, items measuring sleep behavior alterations (items 1–3) were the least informative items, with information being lowest for item 3 (waking too early), second lowest for item 2 (difficulty maintaining sleep), and third lowest for item 1 (difficulty initiating sleep).

## Discussion

This study expanded on previous studies characterizing insomnia complaints among people with EDs by evaluating the frequency of endorsement of sleep behavior alterations and insomnia-related impairment and distress. This study used IRT analysis of the ISI to determine which ISI items were most reflective of severe, likely clinically significant, insomnia and contextualized these findings in light of previous IRT analysis by Morin et al. [35] of the ISI in a diagnosed insomnia disorder population. Our hypothesis that the IRT parameters of the ISI in this sample of people with EDs would be similar to that of the insomnia disorder sample was supported.

Overall, participants in the sample reported high levels of clinically significant insomnia, with the majority reporting a score above the cutoff on the ISI for insomnia case status, which was consistent with prior research that insomnia symptoms are elevated among people with EDs [6, 8, 18, 47]. One reason for this is that insomnia symptoms and ED symptoms likely have a bidirectional relationship, e.g., [15] that results in alterations to cognitive, physiological, and behavioral processes that exacerbate and maintain eating and sleep pathology [1].

This study further clarified the nature of specific insomnia symptoms within an ED sample, converging with work conducted by Kim et al. [18] and Nagata et al. [26] on the prevalence of complaints such as difficulty initiating sleep, maintaining sleep, or waking too early and extending to encompass impairment and distress-related symptoms. Symptom endorsement for all items in the ISI was high, with the most common symptom on the measure (dissatisfaction with sleep) reported by approximately 87% of participants, and even the least common symptom (problems waking too early) endorsed by at least 40% of participants. The symptom endorsement pattern of this measure provides additional insight into frequency and nature of insomnia symptoms among people with ED pathology.

The performance of the ISI among this sample of people with probable EDs was largely similar to that of the IRT conducted by Morin et al. [35]. This suggests that the nature of insomnia symptoms among people with EDs may not significantly differ from those with insomnia disorder. This provides further corroboration to the notion that insomnia disorder may be a distinct diagnosis [48], regardless of comorbidity. However, further research is needed to examine symptom presentation among people with *DSM*-diagnosed EDs, people with insomnia disorder, and those with co-occurring diagnoses.

IRT analyses showed that in the probable ED sample, impairment- and distress-related items on the ISI, and in particular, the item assessing worry/distress about sleep, showed the overall best discriminative capacities and highest item information. In other words, perception of problems or concerns about sleep best distinguished across the range of insomnia severity in this sample. By contrast, items that measured sleep behaviors (e.g., difficulty initiating sleep, difficulty maintaining sleep, and waking up too early) showed poorer overall discriminative capacity. These results converge with the IRT analyses by Morin et al. [35] of the ISI’s performance among people with insomnia disorder, in which they found that difficulty falling asleep and waking up too early showed poor discriminative capacity. However, it should also be noted that in the current study, waking up too early yielded comparably very high ‘difficulty’ parameters for the more severe response categories specifically, suggesting that providers should especially attend to when an individual endorses marked difficulties with early morning awakenings.

In both analyses, sleep behavior alterations were not the best indicators of item discrimination. It is possible that sleep behavior alterations, while essential components of the insomnia diagnosis are relatively non-specific symptoms in most cases. For example, it is possible that some people may have sufficient schedule flexibility that difficulties falling asleep, maintaining sleep, or waking too early can be “compensated” for with sleeping in, going to sleep early, or daytime napping. Thus, the negative consequences of these sleep behavior alterations may be mitigated, resulting in individuals who endorse behaviors, but not impairment or distress, related to insomnia. By contrast, impairment- and distress-related concerns, which reflect the other essential insomnia disorder diagnostic criteria of daytime dysfunction, could represent a more salient indicator of sleep disturbance with a cause rooted more directly in the insomnia latent trait. Simply put, these findings suggest that while alterations in sleep patterns may not always indicate clinically significant sleep problems, the presence of certain aspects of self-reported impairment (especially worry/distress) more likely indicates clinically significant insomnia. An important implication is that impairment and distress items are perhaps the most useful questions for insomnia screening purposes, particularly when ED symptoms co-occur.

## Strengths and limitations

This study has multiple strengths. First, previous research on sleep problems in EDs has frequently used unvalidated or single-item measures of sleep disturbances (e.g., difficulty falling or staying asleep). This study used a well-validated comprehensive measure of insomnia symptom pathology that captured both sleep behavior alterations and impairment and distress. It is the first study to thoroughly characterize symptom-level presentations of insomnia (including distress and impairment) and to contextualize these findings with previous research using IRT to evaluate the latent construct of insomnia.

There are several aspects of this study that limit its generalizability. First, this study examined a population of university students. Consequently, findings may or may not generalize to the general population of adults with EDs. For example, in the university student population, certain sleep complaints may reflect challenges related to university life that are not necessarily related to insomnia. One sleep complaint assessed is satisfaction or dissatisfaction with sleep patterns, which could reflect common sleep issues experienced in this population such as shifting sleep schedules throughout the week. Similarly, students with roommates may be likely to experience difficulties with falling or staying asleep related to living in disruptive sleep environments (e.g., dorms), which may not reflect insomnia pathology.

Second, this study did not have information about co-occurring mental health conditions that are related to insomnia and ED symptoms and to university students or a full validation of the symptoms necessary to evaluate a diagnosis of insomnia disorder (e.g., duration of symptoms, rule-out of physiological effects of substance use). Although insomnia symptoms are among the criteria for major depressive disorder and generalized anxiety disorder, insomnia disorder is a distinct diagnosis [48]. This is particularly relevant as anxiety and depressive symptoms have been found to mediate between insomnia and ED psychopathology [6], as well as between insomnia and binge-eating behaviors [49]. Furthermore, previous research found that controlling for depression attenuated the observed association between eating and sleep pathology [26]. Future studies should include assessments of co-occurring depressive, anxiety, and trauma-related disorders to better understand the clinical presentation of people with EDs and sleep problems and a more rigorous evaluation of insomnia disorder criteria. This would allow researchers to better understand if findings represent insomnia symptom presentation as a correlate of other mental health disorders or insomnia disorder as a potential independent diagnosis.

Third, this sample was relatively racially and ethnically homogenous and had a high proportion of women. More research in racially, ethnically diverse samples is needed as research suggests there are significant elevations in sleep [50] and eating pathology [51] in minoritized racial/ethnic groups, which may impact functioning and contribute to further disparities. Future studies are also needed to recruit and examine insomnia symptoms in people with minoritized gender identities and sexual orientations, who are also more likely to experience elevated ED symptoms [52] and sleep problems [53, 54]. Finally, the use of self-reports to ascertain probable ED diagnosis may have resulted in over- or under-identification of individuals with EDs and mischaracterization of insomnia symptom presentation among people with EDs. Previous studies have found prevalence of probable EDs among university students to be 20–36% using self-report measures [55, 56], with one study finding an additional 20% of students reported symptoms consistent with a possible clinical/subclinical ED [56]. The prevalence of EDs of about 30% observed in this sample is thus consistent with prior literature; however, it is possible our sample may have exhibited selection bias, given that the survey was advertised as being about eating and weight concerns and offered treatment referral resources. Although this method of establishing probable ED diagnosis has been used in previous studies [57], conducting the analyses from this study in a sample of people with an interview-diagnosed ED would increase confidence in findings.

## Conclusions

The current findings add to a growing body of research suggesting clinically significant insomnia symptoms are an important, yet often under-recognized, co-occurrence with ED pathology. Results expand upon prior findings by examining the nature of specific insomnia symptoms, moving beyond complaints such as difficulty initiating sleep, maintaining sleep, or waking early to encompass symptoms associated with impairment and distress. In particular, the study highlights the importance of subjective sleep-related experiences, such as distress and impairment, in identifying clinically notable sleep disturbances among individuals with EDs. Specifically, insomnia assessment items related to sleep behavior alterations, such as difficulty falling asleep or staying asleep, largely failed to differentiate between people high and low in the insomnia trait, especially when compared to items indexing sleep-related distress/impairment. This suggests that research on sleep and EDs that has primarily focused on sleep behavior alterations could potentially overestimate the significance of these changes, while missing out on other important components. Theoretical work has also emphasized the important role of emotional processes in the development and maintenance of disordered-eating behaviors, demonstrating a potential mechanistic alignment between insomnia and EDs. De Young and Bottera [2] recommend integration of their circadian theory of disordered-eating behavior with affect regulation theories of disordered eating to further examine potential links between misalignment of circadian rhythms (e.g., sleep/wake rhythms) and affective processes. The present findings demonstrate the significance of distress and impairment related to insomnia among individuals with EDs and may therefore have implications in future efforts toward integration of theoretical models. Clinically, this study provides additional support for the necessity of Criterion B of insomnia disorder classification (i.e., that the sleep problems must cause “clinically significant distress or impairment in social, occupational, or other important areas of functioning”) among individuals with EDs. Further, this study highlights that, among ED patients, screening measures that emphasize how insomnia symptoms impact individuals may be more informative clinically, and could be evaluated by practitioners using brief assessments. Moving forward, sleep research with individuals with EDs that assesses the subjective experiences of distress and impairment is highly warranted to provide a more complete picture of the experience of insomnia in this population.

This study is the first to examine the properties of a well-validated insomnia symptom measure in a sample of people with transdiagnostic ED pathology (and only the second IRT analysis of the ISI measure in the literature). Future research may build upon these findings to examine associations between specific insomnia symptoms and ED behaviors (e.g., purging, binge eating, restriction) such as through techniques like network analysis [58, 59]. Specifically, this study will facilitate network research by helping identify the most reliable items that can then later be used to test the network structure to further explore associations among ED and insomnia symptoms in future research. Future studies should also explore mechanisms underlying the insomnia-ED association [1, 2, 60] and determine whether leveraging behavioral sleep medicine to intervene on insomnia symptoms may be an adjunctive intervention to help to reduce ED symptoms and improve quality of life among individuals with EDs.

## What is already known on this topic?

Sleep behavior alterations associated with insomnia (e.g., difficulty falling or staying asleep) are reported among people with EDs although there is limited knowledge about the presence of insomnia-related impairment and distress and the frequencies of these symptoms. Furthermore, although previous studies have examined the latent construct of insomnia among people with insomnia disorder, it is unclear how a validated measure of insomnia performs among people with EDs. Specifically, it is unknown if the endorsement of specific insomnia symptoms (e.g., sleep behavior alterations, impairment) is associated with increased insomnia severity in the context of EDs or if sleep behavior alterations and daytime dysfunction are interchangeable in their associations with the latent construct of insomnia.

## What this study adds?

This study characterizes the presence of specific insomnia symptoms (encompassing impairment and distress) among people with EDs and uses psychometric techniques to show that insomnia-related distress and impairment items may have greater utility in assessing the presence of insomnia than sleep behavior alterations, which have been more frequently used in the current literature on EDs and sleep problems.

## Data Availability

Participants of this study did not consent for their data to be shared publicly; thus, supporting data are not publicly available. Individuals who are interested in obtaining the de-identified data should contact the corresponding author after completing a data-use agreement and obtaining local Institutional Review Board approval.
